# Prefrontal and Cerebellar Contributions to Semantic Memory Retrieval

**DOI:** 10.1162/NOL.a.235

**Published:** 2026-03-26

**Authors:** Adam Kubinec, Rastislav Rovný, Igor Riečanský, Martin Marko

**Affiliations:** Department of Behavioural Neuroscience, Institute of Normal and Pathological Physiology, Centre of Experimental Medicine, Slovak Academy of Sciences, Bratislava, Slovakia; Department of Psychiatry, Faculty of Medicine, Slovak Medical University in Bratislava, Bratislava, Slovakia; Department of Applied Informatics, Faculty of Mathematics, Physics and Informatics, Comenius University in Bratislava, Bratislava, Slovakia

**Keywords:** cerebellum, executive functions, inhibition, language, noninvasive neuromodulation, prefrontal cortex, semantic memory

## Abstract

Semantic memory retrieval is essential for language, thought, and adaptive behavior. Although both the prefrontal cortex (PFC) and the cerebellum have been implicated in this function, the role of the PFC remains poorly understood and the contribution of the cerebellum largely overlooked in current neurocognitive models. To address these gaps, we conducted a double-blind, randomized, placebo-controlled experiment in which healthy adults received anodal transcranial direct current stimulation (tDCS) targeting the left lateral PFC, the right posterior cerebellum, or sham stimulation. Participants completed a novel process-sensitive paradigm comprising lexical decision, free-associative (FA; automatic) retrieval, dissociative (DA; controlled) retrieval, and intrusion monitoring, while manipulating response predictability and rule switching. Cerebellar tDCS selectively impaired FA performance, particularly for cues evoking predictable responses, supporting its role in automatic access to overlearned associations. In contrast, prefrontal tDCS disrupted DA performance and increased associative intrusions, implicating the PFC in retrieval inhibition. Importantly, mediation analysis showed that the reduction in DA fluency was largely explained by higher probability of intrusions, indicating a perturbation of proactive inhibitory control that normally prevents irrelevant memory activations from entering working memory. Further exploratory analyses ruled out several alternative accounts of these stimulation effects, underscoring their process specificity. Together, these findings advance models of semantic cognition by demonstrating complementary contributions of the cerebellum to automatic retrieval and of the PFC to inhibitory control over intrusions.

## INTRODUCTION

Semantic cognition encompasses the neural and cognitive mechanisms that support the acquisition, organization, and retrieval of conceptual knowledge, enabling individuals to learn from the statistical structure of multimodal experience and to deploy concepts flexibly in service of adaptive thought and behavior (Kumar, 2021; Lambon Ralph et al., 2017). The human brain stores vast amounts of knowledge about the world, posing a fundamental challenge for cognitive neuroscience to explain how conceptual representations are efficiently accessed and retrieved to meet the demands of dynamic environments and internally generated goals.

Decades of research indicate that memory retrieval arises from a dynamic interplay between automatic and controlled processes, which are thought to rely on differentiable cognitive operations and neural systems (Badre & Wagner, 2002, 2007). Automatic retrieval from semantic memory is a rapid process that draws on well-established associative links, requiring minimal cognitive effort or executive oversight. It is triggered by strong bottom-up activation of representations related to retrieval cues and typically produces overlearned responses, as seen in variants of word association paradigms (Allen et al., 2008; Benedek et al., 2012; Gray et al., 2019; Marko et al., 2024; Marron et al., 2018) and verbal fluency tasks (Marko et al., 2023; Michalko et al., 2023). In contrast, controlled retrieval engages executive processes to guide memory search when automatically activated representations are not aligned with current goals and access to less dominant information is required (Badre & Wagner, 2002). Neuroimaging research has mapped these modes of retrieval onto dissociable neural systems: Automatic retrieval is primarily supported by coordinated activity between the semantic and default mode networks (Marron et al., 2018, 2020; Menon, 2023), whereas the control of retrieval engages a left-lateralized semantic control network, anchored in the left inferior frontal gyrus and extending to dorsal prefrontal and posterior temporal cortices (for reviews, see Chiou et al., 2018, 2023; Lambon Ralph et al., 2017). These findings provide the foundation for contemporary neurophysiological models of semantic cognition, which propose a distributed cortical architecture supporting both automatic and controlled retrieval processes.

Despite the progress in understanding the cortical systems supporting semantic cognition, current models have largely neglected extracortical contributions, most notably those of the cerebellum. The cerebellum, particularly the right posterior lobe, is reciprocally connected with left-lateralized cortical association areas implicated in language and memory (Argyropoulos et al., 2020; Buckner, 2013; King et al., 2019) and shows robust activation during tasks involving semantic processing and retrieval (D'Mello et al., 2017; Gatti et al., 2020; Gilligan & Rafal, 2019; Petersen et al., 1989; Petríková et al., 2023; for reviews, see Marien et al., 2001; Schmahmann, 2010). A compelling view, grounded in its uniform microstructure, posits that the cerebellum supports cognition by constructing internal models through associative learning (Argyropoulos, 2016; Ito, 2008), drawing on sensory and linguistic experience to extract regularities, form habitual associative structures (Gatti et al., 2022), and link discrete “units of thought” into overlearned sequences (Thach, 1998, 2007). Together, these perspectives suggest that the cerebellum is an important hub in semantic cognition that may facilitate automatic semantic retrieval by shaping patterns of conceptual activation through learned associative structure. Despite accumulating evidence, comprehensive tests of this hypothesis are scarce, exposing a critical gap in current theoretical frameworks.

Conversely, although the prefrontal cortex (PFC), especially the left inferior frontal regions, has been consistently implicated in controlled semantic processing and retrieval (Badre & Wagner, 2007; Lambon Ralph et al., 2017), its precise functional contributions remain a matter of ongoing debate. For instance, prior work has linked the left PFC activation to the depth of semantic processing (Nyberg, 2002; Peng et al., 2024), flexible switching (Sohn et al., 2000; Troyer et al., 1998), selection among competing associates or concepts (Badre et al., 2005; Thompson-Schill et al., 1997), and/or their inhibition (Marko, Cimrová, & Riečanský, 2019; Marko & Riečanský, 2021a). Among cognitive control mechanisms, inhibition has been highlighted as especially critical for memory functioning and features prominently in several influential theoretical models (M. C. Anderson & Green, 2001; Apšvalka et al., 2022; Murayama et al., 2014). In the context of goal-oriented memory retrieval, inhibitory control is thought to play a central role in suppressing strongly associated but irrelevant concepts and intrusions (Clark et al., 2016; Kühn et al., 2013), thereby aligning memory outputs with current task demands and enabling coherent as well as flexible cognitive processing. Yet, clarifying whether such prefrontal activity during memory retrieval reflects inhibitory function (and identifying the mechanisms that support it) remains a timely and important avenue for advancing neurocognitive theories of semantic control (for recent reviews, see M. C. Anderson et al., 2025; Wessel & Anderson, 2024).

The present study addressed key gaps in current models of semantic memory by investigating the contributions of the cerebellum and the PFC to conceptual retrieval. To this end, we conducted a double-blind, randomized, placebo-controlled neurostimulation experiment, in which healthy participants performed semantic memory retrieval tasks at baseline and then during anodal transcranial direct current stimulation (tDCS) targeting either the right posterior cerebellum, a region implicated in semantic processes and memory retrieval (Argyropoulos et al., 2020; King et al., 2019), or the left inferior PFC, a key region for controlled semantic processing (Badre & Wagner, 2007; Lambon Ralph et al., 2017). tDCS is a safe, well-tolerated noninvasive brain stimulation technique (Bikson et al., 2016) that can effectively modulate the excitability and functional connectivity of brain regions (Chang et al., 2021; Rahman et al., 2013; Stagg & Nitsche, 2011), influencing a broad range of cognitive functions, including memory, language, and executive control (Brasil-Neto, 2012; Plewnia et al., 2015; Price et al., 2015; Sarkis et al., 2014; Strobach & Antonenko, 2017). Following that, we used tDCS to test two main hypotheses. Our first hypothesis was that the cerebellum supports automatic semantic retrieval by shaping conceptual activation through learned associative structure (Gatti et al., 2022; Petríková et al., 2023), thereby enabling rapid access to overlearned word associations. Accordingly, we predicted that excitatory (anodal) stimulation of the right posterior cerebellum would reduce free-associative (FA) retrieval latencies relative to baseline and that this facilitation would surpass the change observed in the sham group. We also evaluated a second hypothesis that the left PFC supports controlled retrieval by inhibiting habitual associations and suppressing spontaneous intrusions that disrupt goal-directed memory search (Kühn et al., 2013; Marko & Riečanský, 2021a; Volle et al., 2012). Thus, we predicted that excitatory prefrontal tDCS would enhance the speed of dissociative (DA) retrieval from baseline and that this enhancement would be greater compared to that observed in the sham group. To assess these retrieval processes, participants performed a modified version of the Associative–Dissociative Task (ADT; Marko et al., 2024), a paradigm designed to contrast two distinct modes of retrieval: In the FA condition, participants retrieved the first concept that came to mind in response to a cue, thereby engaging automatic, associative retrieval processes, whereas in the DA condition, they were instructed to retrieve a concept that was unrelated to the cue, requiring active suppression of habitual associates, thereby taxing inhibitory control. Accordingly, the ADT offers a significant methodological advantage over classical verbal fluency tasks, enabling a more precise dissociation between automatic and controlled retrieval processes (Marko et al., 2023, 2024), thus providing an appropriate framework for testing the core hypotheses of this study.

Crucially, the present study incorporated several targeted design features to extend prior work and enhance the specificity of inferences regarding the distinct functional roles of cerebellar and prefrontal regions. First, to evaluate whether cerebellar involvement depends on the automaticity and predictability of responses (D'Mello et al., 2017; Gatti et al., 2022), we manipulated the probability that strong and typical associations underlie retrieval. To this end, we varied associative typicality (an item-level property) by including stimuli that evoked either a few dominant associations (high typicality, low selection demands) or many weaker ones (low typicality, high selection demands) in the ADT. This manipulation allowed us not only to test whether cerebellar stimulation selectively modulates the retrieval of overlearned, highly typical associations but also to explore the alternative account that the left PFC supports conceptual selection (i.e., resolving competition among activated concepts) over inhibition per se (Badre et al., 2005; Thompson-Schill et al., 1997). Second, because retrieval conditions in the ADT alternated unpredictably, the task allowed us to probe whether the PFC enables flexible switching between retrieval rules (Sohn et al., 2000). Third, the ADT was modified such that each trial was preceded by a simple lexical decision task (LDT), enabling us to dissociate early lexical processes (i.e., form-based recognition and lexical access; Gold et al., 2006) from subsequent semantic retrieval processes (FA and DA) and, thereby, further enhance the interpretive precision of any stimulation effects. Finally, compared to our prior work (Marko et al., 2024; Marko & Riečanský, 2021a; Petríková et al., 2023), the retrieval task in the present study was extended to assess associative intrusions—spontaneously triggered associations or mental imagery intruding into awareness during DA retrieval and disrupting performance. This measure provided a critical window into the dynamics of inhibitory control, allowing us to distinguish between proactive/early inhibition (preventing intrusions) and reactive/corrective inhibition (suppressing intrusions after they occur; see Braver, 2012; Irlbacher et al., 2014). Accordingly, we reasoned that if the left PFC contributes specifically to proactive inhibitory control, then prefrontal tDCS should reduce the likelihood of associative intrusions during DA trials relative to baseline, with this reduction exceeding that observed in the sham group. In this case, we expected that the effect of prefrontal tDCS on DA retrieval performance would be at least partly mediated by the tDCS-induced changes in intrusion rates (i.e., improved control over automatic associative intrusions should yield faster DA response times [RTs]). Conversely, if the left PFC primarily supports reactive inhibitory control, intrusion rates should remain unaffected by the stimulation. By integrating brain stimulation with a process-sensitive experimental paradigm, this study provides a novel, mechanistically informed account of how cerebellar and prefrontal systems support semantic memory—functions that are foundational for language and higher cognition.

## MATERIALS AND METHODS

### Participants

Based on our previous studies of similar design and outcome measures (Marko & Riečanský, 2021a; Petríková et al., 2023) and power considerations for detecting small- to medium-sized effects (*r* ≥ 0.3 for a simple correlation or *d* > 0.5 for a between-group comparison, at 1 − β = 0.80 and α = 0.05), we estimated that a total sample of approximately 147 participants would be required. Following that, a total of 153 participants were recruited. Four participants were excluded from the sample for noncompliance with task instructions, resulting in a final sample of 149 participants (97 females; mean age = 22.7 years, *SD* = 2.9). All participants were right-handed, as assessed with the Edinburgh Handedness Inventory–Short Form (Veale, 2014; mean score = 90.6, *SD* = 13.5), monolingual native Slovak speakers, with no history of psychiatric disorders, and no current medication. The participants were pseudorandomly assigned to balanced stimulation groups targeting either the left PFC (*n* = 49) or the right cerebellum (*n* = 50) or to a sham stimulation group (*n* = 50). The groups did not significantly differ in the proportion of gender (χ^2^ = 0.987, *df* = 2, *p* = 0.611), age, motivational and affective dimensions, frequency of/distress from psychotic experiences, or verbal fluency measures (see Table 1). The research was conducted in accordance with the Declaration of Helsinki (World Medical Association, 2013) and approved by the institutional review board. All procedures and methods were carried out in accordance with the relevant guidelines and regulations. All participants gave written informed consent and received a financial reward for their participation.

**Table 1.** Summary of demographic characteristics, self-reported measures, and verbal fluency performance by stimulation group.

Measure		Sham (*n* = 50)		PFC (*n* = 49)		CER (*n* = 50)			Group comparison			
		*M*	*SD*	*M*	*SD*	*M*	*SD*		*F*	*df*	*p*	BF_10_
Age		22.9	3.5	22.7	2.9	22.6	2.2		0.09	2, 146	0.915	0.071
Handedness score		91.8	12.3	91.3	14	88.8	14.1		0.72	2, 146	0.487	0.122

State	Arousal	65.6	20.2	64.7	20.4	66	17.8		0.06	2, 146	0.94	0.070
	Motivation	80.5	14.5	81.3	14.2	79.2	17.3		0.24	2, 146	0.789	0.081
	Positive affect	71.1	15.5	69.7	15.5	70.4	16		0.11	2, 146	0.899	0.073
	Stress	17.1	17.6	15.5	15.7	12.2	11.8		1.33	2, 146	0.269	0.204

CAPE	Positive FRQ	1.5	0.2	1.5	0.2	1.4	0.3		0.05	2, 144	0.948	0.070
	Positive DIS	1.8	0.5	1.9	0.5	1.8	0.5		0.16	2, 144	0.851	0.077
	Negative FRQ	2	0.3	2	0.4	2	0.4		0.31	2, 144	0.737	0.087
	Negative DIS	2.4	0.6	2.4	0.5	2.3	0.5		0.41	2, 144	0.667	0.095
	Depressive FRQ	1.9	0.4	2	0.4	2	0.4		0.23	2, 143	0.796	0.082
	Depressive DIS	2.6	0.6	2.6	0.5	2.5	0.6		0.3	2, 144	0.741	0.086

Fluency	Category	43.3	7.4	43	6.5	43.3	7.9		0.04	2, 132	0.965	0.074
	Letter	37.6	7.3	37.6	8.8	36.9	7.5		0.13	2, 139	0.875	0.077
	Restricted	24.9	5.8	26.1	6.9	25.9	7.8		0.41	2, 142	0.663	0.096

*Note*. Sham = sham transcranial direct current stimulation (tDCS); PFC = prefrontal tDCS; CER = cerebellar tDCS; BF_10_ = Bayes factor quantifying the evidence for an effect relative to the null hypothesis; CAPE = Community Assessment of Psychic Experiences; FRQ = frequency; DIS = distress.

### Design and Procedure

**Figure 1.**
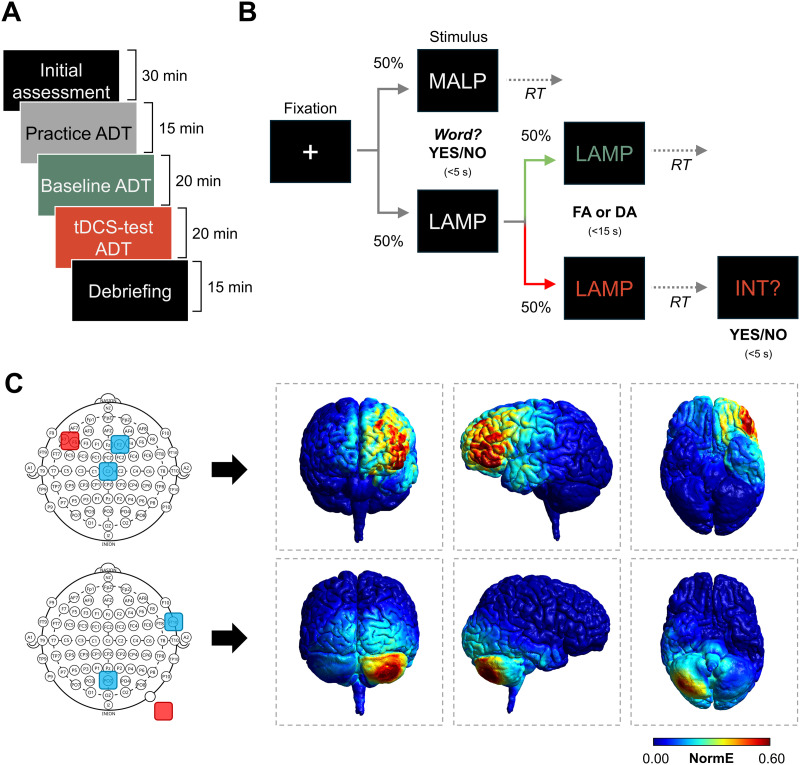
Experimental procedure, task, and transcranial direct current stimulation (tDCS) montages. (A) Experimental procedure with approximate durations. Participants completed initial assessments (questionnaires and verbal fluency tasks), a practice Associative–Dissociative Task (ADT), a baseline ADT block, and a second ADT block during active or sham tDCS, followed by debriefing (tDCS setup and breaks not shown). (B) Trial structure of the modified ADT. Each trial began with a fixation cross and a lexical decision (word vs. pseudoword); word trials proceeded to retrieval: free-associative (FA; green) or dissociative (DA; red), with intrusions (INT) monitored in DA trials. (C) Electrode montages for prefrontal and cerebellar tDCS, along with the corresponding estimated electric field distributions. RT = response time.

The study employed a double-blind, randomized, placebo-controlled experimental design with three main factors: a between-subjects factor, *tDCS* (sham, PFC [prefrontal], CER [cerebellar]), and two within-subject factors, *block* (baseline, tDCS-test) and *retrieval* (FA, DA). Participants completed the sessions individually between 9:00 a.m. and 5:00 p.m. (for an overview, see Figure 1A). Each session began with an initial briefing, which included a short anamnestic interview and the completion of self-report questionnaires, followed by the assessment of verbal fluency. Subsequently, participants were escorted to the main laboratory, where tDCS electrodes were mounted. Participants then completed an extended practice phase, during which they were thoroughly familiarized with the experimental tasks and procedures. After a short break (∼5 min), the baseline ADT measurement was completed. Stimulation was initiated immediately thereafter. Participants first rated any perceived adverse effects of the stimulation and then, after 2 min of full-intensity tDCS, proceeded to complete the second ADT measurement until the stimulation ended (full duration of tDCS was applied even if participants finished the task earlier). At both the beginning and the end of each measurement block, participants reported their current affective and motivational states using dedicated rating scales. Upon completion of the second assessment block, tDCS was terminated, and a short debriefing interview was conducted.

### tDCS

Electrical brain stimulation was delivered using a certified, battery-driven direct current stimulator (DC-STIMULATOR PLUS, neuroConn). One anodal and two cathodal conductive rubber electrodes with a 3 × 3 cm^2^ surface area were attached to the scalp using an electroencephalography (EEG) cap, elastic bands, and conductive electrode paste (Ten20, Weaver and Company). Electrode placement was guided by electric field simulations in SimNIBS (Version 3.2.3; Thielscher et al., 2015) performed on a common template head (i.e., without individualization), using standard quasi-static finite-element tDCS simulations with default tissue conductivities and electrode models. The final montages were selected to maximize the electric field magnitude (normE) within the left inferolateral PFC (PFC tDCS) and right cerebellum (CER tDCS; see Figure 1C). In the PFC group, the anode was centered between F5 and F7, whereas the cathodes were located at the F2 and Cz sites of the International 10–10 system of EEG electrode placement. In the CER group, the anode was centered approximately 2 cm below the PO10 site, and the cathodes were placed at POz and FT10. The stimulation intensity was set to 2 mA (0.22 mA/cm^2^ for anode and 0.11 mA/cm^2^ for cathodes) for both active conditions, which were delivered for 20 min. The stimulation included an additional 60-s ramp-up and ramp-down period. The sham group had the same electrode placement as either of the active conditions (balanced across participants), but the full-intensity current was delivered for only 40 s. The impedance was kept below 10 kΩ. The participants and experimenters were not informed whether real or sham stimulation was applied. During the ramp-up period and the first 2 min of full-intensity tDCS, the participants reported the perceived intensity of adverse effects every 30 s using four visual analog scales (“burning,” “itching,” “pinching,” and “pain,” ranging from 0 = *not at all* to 100 = *very much*). These ratings were subsequently averaged to assess the total level of adverse effects. Overall, stimulation was well tolerated (*M* ± *SE* = 20.1 ± 2.3 for sham, 19.0 ± 2.2 for PFC tDCS, and 22.0 ± 2.2 for CER tDCS), and a one-way analysis of variance (ANOVA) revealed no significant differences in the total score of adverse effects among the experimental groups, *F*(2, 143) = 0.49, *p* = 0.613, η^2^ < 0.01. Finally, at the end of each session, the participants were asked to guess whether sham or real tDCS had been delivered to further evaluate experimental blinding. A chi-square test indicated that the participants were not able to distinguish sham from verum stimulation beyond chance, χ^2^(1, *N* = 149) = 0.57, *p* = 0.450.

### Verbal Fluency

Prior to the main experimental procedure, participants completed a verbal fluency assessment to evaluate baseline lexical-semantic retrieval abilities. This assessment served as a control cognitive measure to ensure comparability between stimulation groups. It consisted of three fluency tasks, each comprising three trials, administered in a fixed order across all participants. On each trial, a stimulus (either a letter or a semantic category) was presented on the computer screen, and participants were instructed to type as many appropriate words as possible within 60 s using the keyboard. A brief practice trial was administered prior to each fluency task to ensure that participants understood the instructions. In the *letter fluency task*, participants generated words beginning with the letters “K,” “D,” and “F” (one letter per trial). In the *category fluency task*, they named items belonging to predefined semantic categories: “Occupations,” “Animals,” and “Electrical appliances.” In the *restricted word fluency task*, participants were asked to generate words that did not contain specific pairs of letters: “K and/or P,” “R and/or S,” and “A and/or E.” All responses were recorded and subsequently evaluated by a blinded trained rater, who scored each item based on preestablished criteria: Words were accepted as correct if they fulfilled the orthographic or semantic requirements of the given trial and were not repetitions, proper nouns, or intrusions. The total score for each fluency task was computed as the sum of correctly generated words across the three trials. A one-way ANOVA on total fluency scores revealed no significant differences between the stimulation groups (see Table 1), indicating that the groups were equivalent in baseline lexical-semantic retrieval performance.

### Semantic Memory Retrieval

The main dependent measures of semantic memory retrieval and retrieval control were assessed using a modified version of the ADT (Marko et al., 2024; see Figure 1B). This computerized paradigm was administered twice—once prior to tDCS (baseline) and once during stimulation (tDCS-test). Each assessment block comprised 40 real words and 40 pseudowords presented in pseudorandomized order. The baseline and tDCS-test featured distinct sets of stimuli, carefully matched on key psycholinguistic and associative characteristics to ensure equivalence across assessment blocks (see Table S1 in Supplementary Materials, available at https://doi.org/10.1162/NOL.a.235).

On each trial, a stimulus appeared in white font at the center of a computer screen, initiating an LDT. Participants had up to 5 s to indicate via keyboard whether the stimulus was a real word or a pseudoword (i.e., a pronounceable but meaningless string of letters) using a two-alternative forced-choice response. Trials containing pseudowords terminated immediately following the lexical decision and did not proceed further. When the stimulus was a real word, the trial continued to the retrieval phase. At this point, the word changed color—either green or red—indicating which retrieval condition participants were to follow. In the FA condition, signaled by green, participants were instructed to retrieve and type a noun that was related to the stimulus as quickly as possible. They were encouraged to respond spontaneously with the first associated word that came to mind, as this condition is designed to probe automatic, associative retrieval with minimal demands on cognitive control (Marko et al., 2023, 2024; Marron et al., 2018). In the DA condition, indicated by red, participants were instructed to generate a noun that was conceptually or associatively unrelated to the stimulus and were informed that producing a related word would be considered an error. This condition aims to elicit controlled retrieval by engaging executive control, specifically the inhibition of dominant associations and memory intrusions (Marko et al., 2024, 2026; Marko & Riečanský, 2021a, 2021b). Each stimulus appeared twice within a block—once in each retrieval condition—resulting in 40 FA and 40 DA trials per assessment block, presented in a counterbalanced order. Participants were allowed to respond within a maximum of 15 s and were instructed to ignore minor grammatical or typing errors and to minimize repeated responses within each testing block as much as possible.

FA retrieval was further manipulated by incorporating two types of stimuli that differed in associative typicality—a stimulus-level characteristic reflecting whether a word normatively elicits one/a few highly dominant/typical responses (high typicality; *n* = 20 words) or a broader range of possible response candidates without a dominant one (low typicality; *n* = 20 words; see Marko et al., 2024, for a psychometric study detailing the procedures used to quantify associative typicality). Furthermore, immediately following each DA trial, participants were asked to indicate whether they had experienced an associative intrusion—i.e., the spontaneous retrieval of an associated word or image in response to the stimulus while attempting to retrieve an unrelated word. Participants responded using a two-alternative forced choice (intrusion absent vs. present) within 5 s. The intrusion ratings were used to differentiate proactive and reactive inhibitory processes during DA retrieval.

Each verbal response was analyzed for retrieval latency, semantic relatedness, and accuracy. Retrieval latency was defined as the time from the onset of the word’s color change to the first keypress of the typed response. Semantic relatedness between each stimulus–response pair was rated by two independent raters, who were blind to participant identity and experimental conditions, using a 6-point ordinal scale (0–5) to reflect increasing levels of associative or conceptual relatedness (see Table S2 in Supplementary Materials). The final relatedness score was calculated as the mean of the two ratings (interrater reliability was high, as indicated by an intraclass correlation coefficient of 0.92). The raters also screened all responses for errors. A response was considered an error if it did not comply with the retrieval condition (i.e., if an FA response received a semantic relatedness score ≤2.5 or a DA response received a score > 2.5), was not a common noun, or was unintelligible.

### Self-Reported Measures

Before the cognitive assessment, participants completed the Community Assessment of Psychic Experiences (CAPE; Stefanis et al., 2002) to evaluate the frequency and distress associated with psychotic-like experiences across three symptom dimensions: positive, negative, and depressive. The CAPE consists of 42 items, each rated on two 4-point ordinal scales—frequency (*never* to *nearly always*) and distress (*not distressed* to *very distressed*). These scores were included as control measures because such subclinical experiences have been previously associated with altered lexical-semantic processing (Hrivikova et al., 2023; Kiang et al., 2010; Merten, 1993) and to verify group equivalence prior to stimulation. One-way ANOVAs revealed no statistically significant differences in CAPE scores among the stimulation groups (see Table 1).

Participants’ psychological state was assessed at the beginning and end of each assessment block using a dedicated self-report scale. The scale comprised 16 items, which were grouped and averaged into four affective and motivational dimensions: *arousal* (“arousal,” “attentiveness,” “fatigue,” “sleepiness”), *motivation* (“commitment,” “eagerness,” “excitement,” “interest”), *positive affect* (“joy,” “ease,” “confidence,” “calm”), and *stress* (“anxiety,” “tension,” “stress,” “nervousness”). Participants rated each item using visual analog scales ranging from 0 (*not at all*) to 100 (*very much*). One-way ANOVAs revealed no statistically significant differences in the four dimensions among the stimulation groups (see Table 1).

### Statistical Analysis

Descriptive analyses of the memory retrieval data revealed outliers (values 1.5 interquartile ranges below Q1 or above Q3) and positive skewness. Therefore, RTs were winsorized (10% two-sided trimming by participant and condition) and analyzed using generalized linear mixed-effects models (GLMMs) with a gamma distribution and identity link function (Lo & Andrews, 2015). For the intrusion reports (a binary dependent measure), a binomial distribution with a logit link function was used. Only correct trials were included in the analyses. The random-effects structure of all GLMMs included random intercepts and slopes for each within-subject fixed effect across participants, as well as random intercepts for stimuli (model specifications are listed in Supplementary Materials). All categorical predictors were effect-coded using sum-to-zero contrasts to enable Type III tests of fixed effects. Model estimation was performed using the bobyqa optimizer with adaptive Gauss–Hermite quadrature, and significance testing was conducted using Type III Wald chi-square tests. Omnibus tests were followed by a priori contrasts that directly operationalized our specific theoretical predictions. As these contrasts constituted a small, predefined set of hypothesis-driven tests, adjustment of their respective *p* values was not applied.

Finally, Bayesian mediation analysis was performed to test the hypothesized mechanistic link between PFC tDCS, associative intrusions, and DA retrieval speed (i.e., whether stimulation-related changes in intrusion probability statistically account for changes in DA latency), which would suggest that the PFC contributes to proactive retrieval inhibition. Bayesian mediation was chosen because it provides a principled framework for estimating the indirect effect (the portion of the tDCS effect attributable to changes in intrusion rates), the direct effect (the remaining effect of tDCS on RT not explained by intrusions), and the total effect (their sum), while allowing these components to be modeled flexibly within a multilevel structure. A Bayesian framework is particularly advantageous in this context because it accommodates different outcome distributions within a unified mediation model (i.e., allowing the binary intrusion measure and the positively skewed RT distribution to be modeled together). To implement this analysis, we used Markov chain Monte Carlo sampling in the brms package (Bürkner, 2017). Two multilevel models were fitted with the package’s default weakly informative priors. The mediator model predicted the binary mediator *intrusion* (absent vs. present) from fixed effects of *block, tDCS*, and their interaction using a Bernoulli likelihood. The outcome model predicted RT using a Gamma distribution with a log link from the same predictors and the mediator. Both models included random intercepts and random slopes for *block* by participant, as well as random intercepts by stimulus. Each model was estimated with four chains, 4,000 iterations per chain (including 1,000 warmup iterations), using a conservative step size adjustment to ensure stable sampling and convergence. Posterior samples from both models were combined to compute mediation parameters following standard Bayesian procedures (Bürkner, 2017). The indirect effect was calculated as the product of the respective posterior coefficients (*a* [tDCS → Intrusion] × *b* [Intrusion → RT]), with direct (*c′*) and total (*c*) effects derived accordingly. All effects were summarized by their posterior means and 95% credible intervals (CrIs). Model convergence was assessed using the potential scale reduction factor ($\hat{R}$), with $\hat{R}$ < 1.01 indicating satisfactory convergence. Effective sample sizes (ESSs) above 1,000 per parameter were taken to indicate sufficient precision and stability of posterior estimates.

## RESULTS

### Paradigm Validation

**Figure 2.**
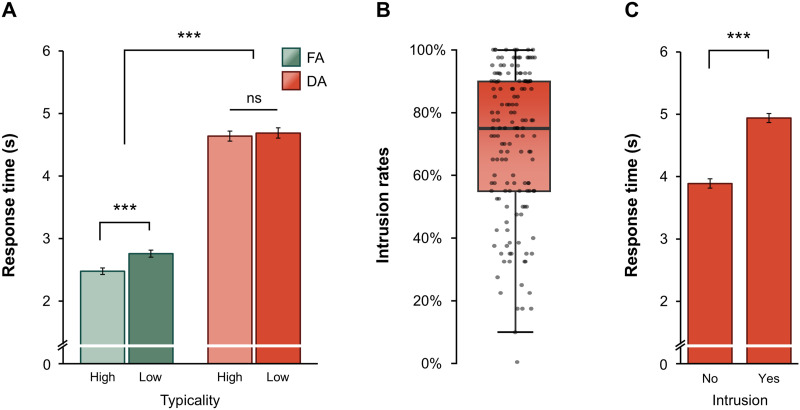
Baseline response times and intrusion rates in the Associative–Dissociative Task. (A) Effects of retrieval condition (free-associative [FA] vs. dissociative [DA]) and associative typicality (high vs. low) on response times. (B) Distribution of intrusion rates in dissociative trials across participants. (C) Effect of associative intrusions on dissociative response times. Error bars represent ±*SE*. ***p < 0.001.

Before testing the main hypotheses, we assessed the validity of the experimental paradigm using baseline data, excluding any tDCS influence. First, a GLMM including *retrieval* (FA vs. DA) and *typicality* (high vs. low) showed a significant interaction between the two fixed factors, χ^2^(1) = 18.05, *p* < 0.001 (Figure 2A). DA responses were generally slower (estimated marginal mean [*EMM*] = 4.66 s, *SE* = 0.072 s) than FA responses (*EMM* = 2.61 s, *SE* = 0.044 s). In FA trials, high-typicality stimuli elicited significantly faster responses than low-typicality stimuli (ΔRT = –0.302 s, *SE* = 0.065 s, *z* = 4.644, *p* < 0.001), whereas no typicality effect was observed in DA trials (ΔRT = –0.045 s, *SE* = 0.074 s, *z* = 0.602, *p* = 0.548). Furthermore, participants reported an average of 70% intrusions during DA trials (see Figure 2B for individual intrusion rates). A GLMM revealed that DA trials with intrusions had significantly higher RTs (*EMM* = 4.94 s, *SE* = 0.073 s) compared to those without intrusions (*EMM* = 3.89 s, *SE* = 0.074 s), χ^2^(1) = 199.65, *p* < 0.001 (Figure 2C). Next, to assess the test–retest reliability of the primary retrieval measures, we calculated mean RTs and intrusion rates separately for the baseline and tDCS-test for each individual in the sham group. Pearson correlation coefficients indicated high reliability in all measures: FA retrieval RTs, *r* = 0.81; DA retrieval RTs, *r* = 0.86; and intrusion rates, *r* = 0.91 (all *p*s < 0.001). Together, the analyses confirmed the validity of the factors included in the modified ADT paradigm, demonstrating robust effects of retrieval condition, associative typicality, and intrusions on RTs, as well as high test–retest reliability of the outcome measures.

### Hypothesis Testing

**Figure 3.**
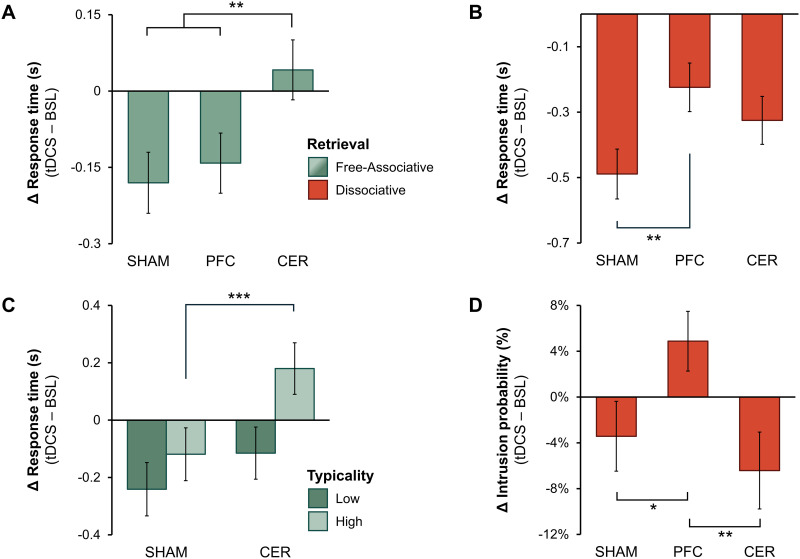
Effects of transcranial direct current stimulation (tDCS) on retrieval measures. All panels display the change in retrieval measures, calculated as tDCS-test minus baseline (BSL), with negative values indicating improved performance. (A) The change in free-associative (FA) retrieval (including low- and high-typicality trials). (B) The change in dissociative (DA) retrieval performance. (C) The change in FA retrieval, assessed separately for low- and high-typicality trials. (D) The change in the model-estimated probability of associative intrusions during DA trials. SHAM = sham tDCS; PFC = prefrontal tDCS; CER = cerebellar tDCS. Error bars represent ±1 *SE*. *p < 0.05, **p < 0.01, ***p < 0.001.

The effects of tDCS on RT in the ADT were analyzed using a GLMM including the factors *block* (baseline vs. tDCS-test), *tDCS* (sham, PFC, CER), and *retrieval* (FA vs. DA). The model revealed a significant three-way interaction among these factors, χ^2^(2) = 11.45, *p* = 0.003 (see Table S3 in Supplementary Materials), indicating that changes in retrieval performance varied across stimulation groups and retrieval conditions. To clarify this complex interaction, we followed up with a series of a priori custom contrasts. The first series of contrasts was conducted to evaluate the hypothesis regarding the changes (tDCS-test vs. baseline) in FA retrieval across the tDCS groups. The results indicated that CER tDCS significantly interfered with FA retrieval compared to the other groups (see Figure 3A). Namely, in the sham group, mean FA retrieval RT significantly decreased from baseline to tDCS-test (ΔRT = −0.180 s, *SE* = 0.060 s, *z* = 3.005, *p* = 0.003), as it did in the PFC group (ΔRT = −0.142 s, *SE* = 0.059 s, *z* = 2.396, *p* = 0.017; see Table 2). In contrast, mean RT in the CER tDCS group slightly increased in tDCS-test compared to the baseline assessment (ΔRT = +0.042 s, *SE* = 0.059 s, *z* = 0.705, *p* = 0.481). Importantly, the change in RT in the CER group differed significantly from both the sham group (ΔRT = +0.222 s, *SE* = 0.068 s, *z* = 3.280, *p* = 0.001) and the PFC group (ΔRT = +0.183 s, *SE* = 0.067 s, *z* = 2.741, *p* = 0.006). The difference between sham and PFC groups was not significant (ΔRT = +0.039 s, *SE* = 0.068 s, *z* = 0.570, *p* = 0.569). The second series of contrasts was conducted to examine the changes (tDCS-test vs. baseline) in DA retrieval across the tDCS groups. The results indicated that DA retrieval was significantly modulated by PFC tDCS, showing a disruptive effect compared to the sham group (see Figure 3B). Specifically, mean RTs decreased from baseline to tDCS-test in all groups: the sham group (ΔRT = –0.489 s, *SE* = 0.076 s, *z* = 6.420, *p* < 0.001), the PFC group (ΔRT = –0.224 s, *SE* = 0.074 s, *z* = 3.013, *p* = 0.003), and the CER group (ΔRT = –0.325 s, *SE* = 0.073 s, *z* = 4.437, *p* < 0.001; see Table 2). However, the decrease in RTs was significantly smaller in the PFC group compared to the sham group (ΔRT = +0.265 s, *SE* = 0.094 s, *z* = 2.823, *p* = 0.005), indicating a negative effect of PFC tDCS on DA retrieval. No significant differences were observed between the CER group and the sham group (ΔRT = +0.164 s, *SE* = 0.093 s, *z* = 1.761, *p* = 0.078) or between the PFC group and the CER group (ΔRT = +0.101 s, *SE* = 0.092 s, *z* = 1.105, *p* = 0.269).

**Table 2.** Estimated marginal means for Associative–Dissociative Task measures by stimulation group at baseline and during transcranial direct current stimulation (tDCS).

Measure	tDCS group	Baseline		tDCS-test		Change (tDCS – baseline)		
		*M*	*SE*	*M*	*SE*	Δ	*SE*	*p*
Free-associative RT (s)	Sham	2.79	0.061	2.61	0.062	−0.18	0.060	0.003
	PFC	2.57	0.061	2.43	0.062	−0.14	0.059	0.017
	CER	2.50	0.060	2.54	0.061	0.04	0.059	0.481

Dissociative RT (s)	Sham	4.97	0.107	4.48	0.106	−0.49	0.076	<0.001
	PFC	4.55	0.106	4.33	0.107	−0.22	0.074	0.003
	CER	4.53	0.105	4.21	0.105	−0.33	0.073	<0.001

Intrusions (%)	Sham	77.8	4.07	74.4	5.21	−3.43	3.05	0.261
	PFC	77.0	4.22	81.9	4.14	4.88	2.61	0.062
	CER	75.5	4.32	69.1	5.77	6.42	3.36	0.056

*Note*. RT = response time; Sham = sham tDCS; PFC = prefrontal tDCS; CER = cerebellar tDCS.

Furthermore, to examine whether associative typicality moderates the effect of tDCS on FA retrieval, a GLMM was conducted, with *block* (baseline vs. tDCS-test), *tDCS* (sham, PFC, CER), and *typicality* (high vs. low) as fixed factors. The model indicated a significant three-way interaction, χ^2^(2) = 7.36, *p* = 0.025 (see Table 3 and Table S4 in Supplementary Materials). Follow-up contrasts revealed that in high-typicality trials, FA performance was significantly impaired by CER tDCS relative to sham (ΔRT = +0.299 s, *SE* = 0.084 s, *z* = 3.539, *p* < 0.001), whereas in low-typicality trials, the effect was smaller and nonsignificant (ΔRT = +0.126 s, *SE* = 0.089 s, *z* = 1.417, *p* = 0.157; see Figure 3C). In line with previous analyses, the PFC group did not differ significantly from the sham group in FA retrieval, regardless of the typicality level (*p* > 0.457).

**Table 3.** Estimated marginal means for free-associative response times by associative typicality and stimulation group at baseline and during transcranial direct current stimulation (tDCS).

Typicality	tDCS group	Baseline		tDCS-test		Change (tDCS – baseline)		
		*M*	*SE*	*M*	*SE*	Δ	*SE*	*p*
Low	Sham	2.92	0.085	2.68	0.082	−0.24	0.093	0.010
	PFC	2.69	0.084	2.52	0.082	−0.18	0.092	0.056
	CER	2.70	0.083	2.59	0.082	−0.12	0.092	0.210
								
High	Sham	2.67	0.078	2.55	0.079	−0.12	0.091	0.193
	PFC	2.47	0.077	2.35	0.078	−0.12	0.090	0.168
	CER	2.33	0.076	2.51	0.079	0.18	0.090	0.045

*Note*. Response times are in seconds. Sham = sham tDCS; PFC = prefrontal tDCS; CER = cerebellar tDCS.

Finally, we examined the effects of tDCS on the probability of associative intrusions during DA trials using a GLMM, with *block* (baseline vs. tDCS-test) and *tDCS* (sham, PFC, CER) as fixed effects. The model revealed a significant *block* × *tDCS* interaction, χ^2^(2) = 11.34, *p* = 0.003 (see Table S5 in Supplementary Materials). Follow-up custom contrasts showed a slight, nonsignificant decrease in intrusion probability from baseline to tDCS-test in the sham group (Δ$\hat{p}$ = −3.43%, *SE* = 3.05%, *z* = 1.124, *p* = 0.261) and the CER group (Δ$\hat{p}$ = −6.42%, *SE* = 3.36%, *z* = 1.909, *p* = 0.056). In contrast, intrusion probability marginally increased in the PFC group (Δ$\hat{p}$ = +4.88%, *SE* = 2.61%, *z* = 1.868, *p* = 0.062; see Table 2). Crucially, the tDCS-test vs. baseline change in intrusion probability in the PFC group was significantly different from that in both the sham group (Δ$\hat{p}$ = +8.32%, *SE* = 3.41%, *z* = 2.437, *p* = 0.015) and the CER group (Δ$\hat{p}$ = +11.31%, *SE* = 3.64%, *z* = 3.110, *p* = 0.002), indicating that PFC stimulation led to an increased probability of reporting intrusions (see Figure 3D). The difference between CER and sham groups was not statistically significant (Δ$\hat{p}$ = −2.99%, *SE* = 3.88%, *z* = 0.770, *p* = 0.441).

### Bayesian Mediation Analysis

To examine whether the negative effect of PFC tDCS on DA retrieval was mediated via the observed increase in the probability of intrusions, we conducted a Bayesian moderated mediation analysis including two multilevel models (see Tables S6 and S7 in Supplementary Materials). The *mediator model* included the main effects of *block* (baseline vs. tDCS-test), *tDCS* (sham vs. PFC), and their interaction to predict the occurrence of intrusion. The model showed that the *block* × *tDCS* interaction credibly predicted the probability of intrusions (β = 0.52, 95% CrI [0.11, 0.93], $\hat{R}$ = 1.00; ESS = 7,147); this translates to an increase in the probability of intrusions by approximately 10% due to PFC tDCS. The outcome model predicted DA RT using the main effects of *block, tDCS*, their interaction, and the binary mediator (*intrusion*). The model revealed a robust effect of intrusion on RT (β = 0.24, 95% CrI [0.22, 0.26], $\hat{R}$ = 1.00; ESS = 25,043), translating to an RT increase of approximately 1.29 s when an intrusion occurred. Importantly, the effects of *tDCS* (β = −0.08, 95% CrI [−0.18, 0.02]) and its interaction with *block* (β = 0.04, 95% CrI [−0.02, 0.09]) were not credibly different from zero. Together, the mediation analysis indicated a credible indirect effect (*a* × *b* = 0.12, 95% CrI [0.03, 0.22]), suggesting that the interfering effect of PFC tDCS on DA RT was mediated by increased occurrence of associative intrusions. This indirect pathway corresponds to an estimated RT increase of approximately 0.63 s. In contrast, the direct effect was not credibly different from zero (*c′* = 0.04, 95% CrI [−0.02, 0.09]), corresponding to a small RT increase of about 0.17 s. These results thus indicate that the influence of tDCS on DA RT was primarily mediated by increased probability of intrusions, which accounted for approximately 78% of the total effect (see Table S8 in Supplementary Materials).

### Exploratory Analyses

To further evaluate the specificity of the observed tDCS effects on semantic retrieval performance, three additional analyses were conducted (for more details, see Tables S9–S11 in Supplementary Materials). We first explored the possible role of the PFC in conceptual selection (i.e., in resolving competition among simultaneously activated concepts in memory). A control GLMM was estimated, which included the additional fixed factor *typicality* (high typicality = low selection demands; low typicality = high selection demands), along with *block* (baseline vs. tDCS-test), *tDCS* (sham, PFC), and *retrieval* (FA vs. DA). The results indicated that the selection demands did not significantly moderate the effects of PFC tDCS on FA and/or DA retrieval latencies, χ^2^(1) < 1.94, *p* > 0.163. Second, to evaluate the potential role of the PFC in rule switching, we introduced a post hoc factor *switch* (yes vs. no), derived from the trial sequence (see Supplementary Materials). The GLMM with retrieval latency as the dependent measure revealed no significant main effect of switching, χ^2^(1) = 1.59, *p* = 0.207, nor any significant interaction between this factor and tDCS across blocks, χ^2^(1) = 1.16, *p* = 0.282. The final analysis tested whether tDCS influenced RTs in the LDT. The GLMM included fixed effects of *block* (baseline vs. tDCS-test), *tDCS* (sham, PFC, CER), *word type* (word vs. non-word), and their interactions. The results showed no significant interaction effects involving tDCS on RTs, χ^2^ < 3.97, *p* > 0.138.

## DISCUSSION

The present study investigated the neurocognitive mechanisms of semantic memory by applying excitatory tDCS targeting the right cerebellum or the left PFC, while healthy individuals completed the ADT, a novel retrieval paradigm designed to probe automatic (FA) and controlled (DA) retrieval functions. FA retrieval was markedly faster than DA retrieval, consistent with the notion that it relies on overlearned memory structures and, hence, is more automatic (Logan, 1988; Marko, Michalko, & Riečanský, 2019). In contrast, DA retrieval—entailing the generation of responses unrelated to the cue—was substantially slower (see Figure 2), reflecting the cognitive demands on inhibitory control required to suppress stimulus-driven activations and prepotent responses (Allen et al., 2008; M. C. Anderson, 2003; Marko et al., 2023, 2024; Volle et al., 2012). To further manipulate the automaticity of retrieval, we varied the associative typicality of stimuli and showed that FA latency is shorter for items that evoke few but highly dominant responses, compared to those with a broader, weaker associative structure (J. R. Anderson, 1983; Marko et al., 2024). This finding substantiates the core assumption that dominant, overlearned associations are retrieved more efficiently, most likely due to their inherent predictability and reduced competition during selection (Badre et al., 2005; Badre & Wagner, 2007; Thompson-Schill et al., 1997). Importantly, the present study offers novel evidence that DA retrieval is frequently accompanied by associative intrusions (i.e., associations and/or mental images that spontaneously intrude into awareness despite task instructions). Such intrusions were reported in approximately 70% of DA trials and exerted a markedly disruptive influence on task performance. This effect aligns with existing theoretical accounts and constitutes a key empirical proof of concept that efficient DA performance depends critically on the recruitment of inhibitory mechanisms suppressing automatic, stimulus-driven activations to sustain coherent, goal-directed memory retrieval (Marko et al., 2026; Marko & Riečanský, 2021a, 2021b). Taken together, these findings establish the ADT as a process-sensitive and psychometrically robust paradigm, capable of isolating key components of semantic memory retrieval (retrieval fluency, inhibition, and intrusion susceptibility), and thus provide a powerful tool for mechanistic investigations into the neurocognitive architecture of semantic memory.

### Cerebellum and Automatic Retrieval From Semantic Memory

Accumulating evidence implicates the cerebellum in a wide range of cognitive processes—including memory and language—yet its contribution to semantic cognition remains poorly understood and largely overlooked in contemporary neurocognitive models (Jefferies et al., 2020; Jung & Lambon Ralph, 2023; Lambon Ralph et al., 2017; also see Wang et al., 2025). To address this critical gap, we modulated the right posterior cerebellar activity with anodal tDCS during the ADT, resulting in a selective disruption of FA performance (Figure 3A). Despite its counterintuitive direction (a point discussed in more detail below), this finding supports the hypothesis that the right cerebellum is an integral component of the neurocognitive system underpinning semantic memory and, more specifically, highlights its involvement in automatic retrieval processes. In line with this, finer-grained analysis showed that CER tDCS disproportionately impaired FA retrieval for cues with strong, dominant associations (Figure 3C). These items are typically produced rapidly (Figure 2A) due to extensive prior exposure, relying heavily on automatized access to overlearned associative structures and demanding little selection or inhibitory control (Desmond et al., 1998; Marko et al., 2024).

Moreover, the broader pattern of results allows for a more precise delineation of the cognitive processes that this brain region may support. First, given the separation of the LDT and the ADT in our paradigm and the absence of any detectable effects on lexical decision latency (Bongaerts et al., 2022), the observed disruption of FA performance is unlikely to originate from the modulation of LDT-related processes, such as form-based word recognition or early lexical access. Instead, these effects appear to emerge at later processing stages (i.e., after the stimulus is recognized as a word), including activation spreading (Argyropoulos & Muggleton, 2013; Gilligan & Rafal, 2019), associative search through semantic memory, and concept recovery (Gatti et al., 2020; Petríková et al., 2023). Second, the lack of CER tDCS effects on DA retrieval measures suggests that the cerebellum selectively supports semantic search and retrieval processes that rely on overlearned associative structures (Gatti et al., 2022; Petríková et al., 2023). Indeed, because naming unrelated words is not a natural retrieval mode encountered in everyday situations, this process does not become automatized through repeated practice, possibly limiting cerebellar involvement (Koziol et al., 2014). Moreover, although both FA and DA retrieval share certain common processes, DA performance depends critically on inhibitory mechanisms that actively suppress related concepts and dominant associates (Marko et al., 2023) and, thus, likely recruits distinct neural systems (particularly the PFC, discussed in more detail below; Allen et al., 2008; Collette et al., 2001; Volle et al., 2012). Taken together, the present findings indicate that the right cerebellum is a key component of the neurocognitive system supporting semantic memory, enabling the rapid retrieval of overlearned associations.

Why is the cerebellum important for such associative processes, and what mechanisms support them? The cerebellum, long associated with motor control (Manto et al., 2012), is now recognized as a domain-general computational hub whose uniform microcircuitry and broad connectivity with association cortices support diverse cognitive functions (Buckner, 2013; King et al., 2019). It is thought to construct internal models (adaptive predictive representations) through associative learning (Argyropoulos, 2016; Ito, 2008; Moberget et al., 2014), optimizing behavior by detecting the statistical structure of repeatedly co-occurring sensory and linguistic events (Argyropoulos et al., 2011; Gatti et al., 2020, 2022). Such computations are well suited to shaping the temporal and associative structure of mental events, enabling the formation of stable conceptual structures (Gatti et al., 2020; Lesage et al., 2016) and the automation of cognitive routines (Ito, 2008; Mariën et al., 2014; Sokolov et al., 2017; Thach, 2007). Importantly, these operations align closely with current models of semantic representation, which hold that organized conceptual structures are distilled from lifelong sensory and linguistic experiences through learning the statistical structure of our multimodal inputs (Bi, 2021; Lambon Ralph et al., 2017; Patterson et al., 2007). While several frameworks, including network-based and distributional approaches, account for how conceptual structures emerge (see Kumar, 2021), the instance theory of semantic memory offers a particularly direct mechanistic parallel. It proposes that knowledge arises from accumulated episodic traces that, upon retrieval, are activated by cues and jointly produce a weighted activation pattern (the *echo*) whose overlapping content yields generalizable meaning and associative structure (Jamieson et al., 2022; Logan, 1988). Consistent with the proposed role of the cerebellum, instance theory holds that with repeated exposure, retrieval becomes progressively automatized: Effortful search is replaced by direct access to the most similar stored instances, enabling rapid and cue-sensitive retrieval of overlearned associations that emerge from prior episodic traces (Jamieson et al., 2022; Logan, 1988). Embedding the present findings within these theoretical perspectives, we propose that the cerebellum optimizes associative structures through learning, refining them into compact, prediction-ready patterns that modulate cortical activations within the semantic representation network. This cerebro–cerebellar integration enables semantic retrieval that is fast, precisely tuned to the current cue, and adaptively biased toward the conceptual patterns most reinforced by prior experience.

### PFC and Controlled Retrieval From Semantic Memory

The PFC is widely recognized as a core component of the neural architecture supporting controlled retrieval from semantic memory (Lambon Ralph et al., 2017). Whereas automatic retrieval is driven by strong cue-based activations, controlled retrieval is recruited when relevant information must be selectively accessed or irrelevant activations suppressed to meet current goals (Badre et al., 2005; Badre & Wagner, 2002). This capacity is attributed to a left-lateralized “semantic control network,” anchored in the inferior frontal gyrus, which exerts top-down influence over semantic representation systems (Chiou et al., 2018, 2023; Jackson et al., 2021). Although there is consensus on the general role of the left PFC in semantic control, its functional involvement remains debated. Competing accounts variously propose that it inhibits prepotent but task-irrelevant representations (M. C. Anderson et al., 2025; Marko & Riečanský, 2021a; Wimber et al., 2015), selects among concurrently activated alternatives (Badre et al., 2005; Moss et al., 2005; Thompson-Schill et al., 1997), or deepens semantic search and processing (Nyberg, 2002; Peng et al., 2024; Wagner et al., 2001). The present experiment addressed these alternatives by imposing demands on inhibitory control and selection over automatic associations and by quantifying intrusion susceptibility during tDCS targeting the left PFC.

We found that PFC tDCS disrupted DA retrieval. Although a facilitatory effect was anticipated (e.g., see Marko & Riečanský, 2021a), this outcome is nevertheless consistent with the view that the left PFC plays a central role in retrieval control (M. C. Anderson et al., 2025; Jin & Maren, 2015; Lambon Ralph et al., 2017). The absence of any effect on FA retrieval indicates that this disruption does not arise from a general modulation of retrieval ability or mechanisms supporting automatic retrieval (Marko et al., 2023). Also, exploratory analyses revealed no interaction between the effects of PFC tDCS and associative typicality (a stimulus-level property that shapes selection demands and response competition; J. R. Anderson, 1983; Marko et al., 2024) or rule switching (derived post hoc from trial sequences) in either retrieval task. The lack of sensitivity of PFC tDCS to selection demands is not in line with the alternative explanation that the observed impairment in DA production reflects a reduced capacity to resolve competing responses—a function typically attributed to prefrontal activity (Badre et al., 2005; Ridderinkhof et al., 2004; Thompson-Schill et al., 1997) but psychometrically distinct from inhibition (Bender et al., 2016). Likewise, rule switching neither affected retrieval latencies nor moderated the tDCS-induced changes in latency, indicating that the observed PFC modulation is independent of executive switching demands. Furthermore, given evidence that the left PFC supports depth of semantic processing (Nyberg, 2002; Peng et al., 2024), one could argue that the stimulation enhanced semantic encoding, thereby amplifying cue-driven activations, increasing intrusions, and slowing DA retrieval. Although this interpretation is consistent with the observed pattern of results, it is unlikely that tDCS globally biased processing toward stronger semantic activation, since such a shift would have also facilitated FA retrieval, where no effect was found. By contrast, the inhibitory account is more directly aligned with the present task demands, explains why the disruption was specific to DA retrieval, and avoids positing an unspecific deepening of semantic processing that paradoxically impairs performance. Thus, the present evidence implicates the PFC in suppressing irrelevant representations that would otherwise intrude upon and disrupt coherent cognition, supporting the interpretation that inhibitory control (specifically the suppression of prepotent retrieval candidates) was the principal function modulated by the prefrontal stimulation.

**Figure 4.**
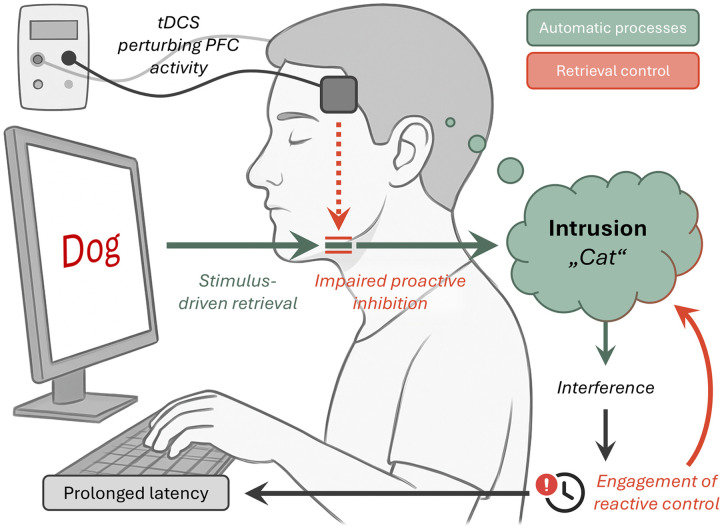
Proposed mechanism of disrupted proactive retrieval control during prefrontal stimulation. Upon stimulus presentation, automatic retrieval rapidly activates associated representations in memory. When such activations are irrelevant, a proactive inhibitory mechanism normally attenuates or halts their entry into working memory, preventing intrusions. This gating function, supported by the left prefrontal cortex (PFC), was perturbed by transcranial direct current stimulation (tDCS), weakening control over the automatic semantic drive and allowing associative intrusions to emerge. Once intrusions enter working memory, they generate interference and trigger reactive control processes. Reactive suppression can expel inappropriate candidates and reduce their short-term accessibility, but this corrective process is time consuming, ultimately leading to delayed responses.

Crucially, the present findings provide novel evidence elucidating a candidate mechanism through which the left PFC may contribute to controlled retrieval. In the present paradigm, we assessed associative intrusions (for a similar approach, see Nardo & Anderson, 2024), a clinically relevant phenomenon linked to PFC functioning (Ahmari & Rauch, 2022; Kühn et al., 2013), which here served as a diagnostic marker of the type and temporal mode of control engaged (Braver, 2012). The reliable increase in intrusion probability during DA trials under PFC tDCS suggests a disruption of proactive control (i.e., an early-stage mechanism that prevents task-irrelevant, stimulus-driven associations/intrusions from gaining representational dominance). By contrast, if reactive inhibitory control (the suppression of intrusions after they occur) had been impaired, intrusion probability would have remained unchanged. Consistent with this reasoning, mediation analysis revealed that the tDCS-induced decline in DA fluency was largely mediated by the heightened likelihood of intrusions. This finding indicates that stimulation specifically weakened proactive inhibition, thereby increasing the susceptibility of working memory to irrelevant associations and obstructing the retrieval of an unrelated word (see Figure 4).

Two—not mutually exclusive—mechanisms may underlie proactive inhibitory control in semantic retrieval. The first is *process-level* inhibition, which interrupts or attenuates cue-driven retrieval, thus preventing related representations from becoming activated. This function could be implemented by fronto-subthalamic-basal ganglia circuitry that modulates thalamocortical drive, thereby preempting downstream representational activation and forestalling automatic associations (Wessel & Anderson, 2024). The second is *representational-level* inhibition, in which retrieval is initiated but stimulus-evoked activity within memory networks (e.g., medial temporal lobe structures and/or semantic “spokes”; Jackson et al., 2021; Patterson et al., 2007) is suppressed before reaching the threshold for conscious access. Such targeted suppression, potentially mediated by local GABAergic interneurons, could silence memory activations within these networks, degrading related memory representations before or even after they are consciously retrieved (M. C. Anderson et al., 2025; Jung et al., 2025). Although both mechanisms may contribute, the process-level gating aligns most closely with proactive control dynamics, whereas representational-level inhibition may more often reflect a reactive operation, engaged when activations in memory circuits become strong enough to require active purging (Levy & Anderson, 2012). Determining whether proactive control in semantic retrieval relies primarily on process-level gating, representational suppression, or their interaction remains a critical goal for future causal and temporally precise investigations.

### Limitations and Future Directions

The present study has several limitations. First, the effects of anodal tDCS observed here diverged from prior findings (see Marko & Riečanský, 2021a; Petríková et al., 2023), with CER tDCS producing disruptive aftereffects rather than facilitating FA retrieval and PFC tDCS impairing rather than enhancing DA retrieval. These discrepancies most likely stem from differences in stimulation montages. Variations in electrode placement, number, and size inevitably reshape the spatial distribution and intensity of neural polarization, thereby altering the effects of tDCS (Bikson et al., 2012; Miranda et al., 2018). Compared to the previous studies using these retrieval measures, our cerebellar montage induced two- to threefold stronger and more localized polarization within the right posterior lobe, whereas the prefrontal montage more selectively targeted inferior and mid-PFC regions, avoiding temporal–parietal areas. At the same time, the present montage may have influenced medial PFC regions, including the anterior cingulate cortex, and potentially subcortical structures, which are also implicated in inhibitory control (M. C. Anderson et al., 2025). Importantly, we suspect that the interfering effects of tDCS stem from the substantially higher excitatory currents induced in the targeted regions compared to the previous studies. Excessive cortical excitation poses a risk of dysfunction and is thought to trigger compensatory homeostatic mechanisms that counteract overexcitability, which can paradoxically suppress rather than enhance neural functioning (Monte-Silva et al., 2013; Staley, 2015; also see Turrigiano, 2011). Relatedly, cathodal/inhibitory stimulation can increase excitability (Batsikadze et al., 2013; Moliadze et al., 2015), further highlighting that tDCS effects follow a nonlinear dose–response profile shaped by homeostatic regulation. Furthermore, because healthy young adults typically operate on a near-optimal level of prefrontal functioning, the stimulation may disrupt this equilibrium, whereas populations with suboptimal control (e.g., older adults or clinical groups) could respond differently, potentially benefiting from such interventions. Thus, variability across stimulation protocols, together with the nonlinear response function, underscores the need for future studies to integrate tDCS with neuroimaging to precisely map the circuits engaged during stimulation and to determine whether behavioral effects arise from localized polarization or from broader network-level adaptations. Relatedly, individualized tDCS protocols are needed to improve the reliability and consistency of stimulation outcomes (Polanía et al., 2018; Van Hoornweder et al., 2025). Future work incorporating subject-specific head models and more advanced current flow modeling (including characterization of inward versus outward field components and field orientation relative to cortical microstructure) may enable more precise targeting of the intended neural circuits and, thereby, enhance the robustness of stimulation effects.

Second, the design of the retrieval paradigm also differed from previous studies. Earlier experiments required chained-associative and DA production (Marko et al., 2023), which may bias participants toward proactive control strategies by reinforcing a consistent retrieval rule across successive trials. In contrast, the present paradigm required discrete responses under randomized conditions, reducing predictability and likely shifting the balance from proactive to reactive modes of control (Mäki-Marttunen et al., 2019; Sohn et al., 2007). Nevertheless, although this difference could explain variations in effect sizes, it is unlikely to account for the observed reversal of stimulation effects. To address this, future research should systematically vary the balance between proactive vs. reactive control in retrieval and examine how these manipulations interact with neural modulation.

Third, although our mediation analysis offered mechanistic insights by linking PFC stimulation, intrusions, and DA fluency, it remains correlational, and the causal structure of these processes cannot be established. Future studies should therefore employ a more suitable adaptation of the paradigm and time-sensitive neural markers, such as EEG or MEG, to test the temporal dynamics of inhibition and intrusion control and to establish causal chains with greater confidence.

Finally, our measure of associative intrusions relied on self-report, which, while psychometrically robust and highly predictive of behavioral disruption, remains inherently subjective. Intrusion ratings are valuable in capturing participants’ internal experience, but complementary objective indices (such as eye tracking, pupillometry, or electrophysiological markers of interference) could strengthen the assessment of intrusions and their dynamics. Incorporating such multimodal markers in future studies would allow for a more precise characterization of how intrusions emerge and how inhibitory control is deployed to manage them.

### Conclusions

The present study extends current models of semantic cognition by providing process-specific evidence for the complementary contributions of cerebellar and prefrontal regions—the cerebellum in supporting the automatic retrieval of overlearned associations and the PFC in exerting proactive inhibitory control over intrusions during controlled retrieval. By demonstrating that these systems constrain semantic activation in distinct yet complementary ways, our findings advance understanding of the neurocognitive architecture of memory. Despite these promising insights, the present results also revealed disruptive rather than facilitatory effects of stimulation, underscoring the need to identify optimal stimulation parameters. Future work should systematically explore dosage, electrode montages, and individualized protocols, ideally in combination with neuroimaging methods, to clarify the neural mechanisms of tDCS and to establish protocols capable of enhancing, rather than impairing, memory functioning. Once such parameters are optimized, the translational potential becomes possible. Cerebellar-targeted interventions may offer new opportunities for improving automatic semantic processing and retrieval in individuals with memory or language deficits. In parallel, prefrontal-targeted approaches could provide means to modulate inhibitory control and reduce intrusive thoughts, a hallmark of conditions such as obsessive–compulsive disorder, post-traumatic stress disorder, depression, and anxiety. Finally, the present study demonstrates the utility of the ADT as a process-sensitive paradigm that isolates automatic retrieval, selection demands, inhibitory control, and intrusion dynamics within a single framework, offering a powerful tool for refining mechanistic models of semantic cognition. Taken together, these contributions move beyond descriptive accounts of semantic memory to identify interactions among specific cognitive operations and their neural substrates.

## Acknowledgments

We thank František Michal Sebestyén and Barbora Michalková for help with data collection.

## Funding Information

Martin Marko, Agentúra na Podporu Výskumu a Vývoja (https://dx.doi.org/10.13039/501100005357), Award ID: APVV-23-0145. Martin Marko, Agentúra Ministerstva Školstva, Vedy, Výskumu a Športu SR (https://dx.doi.org/10.13039/501100003194), Award ID: 2/0052/23. Igor Riečanský, Agentúra Ministerstva Školstva, Vedy, Výskumu a Športu SR (https://dx.doi.org/10.13039/501100003194), Award ID: 2/0067/25. Adam Kubinec, DoktoGrant, Award ID: APP0598.

## Author Contributions

**A.K.:** Conceptualization; Investigation; Project administration; Writing – original draft; Writing – review & editing. **R.R.:** Investigation; Writing – original draft; Writing – review & editing. **I.R.:** Data curation; Supervision; Writing – original draft; Writing – review & editing. **M.M.:** Conceptualization; Data curation; Formal analysis; Funding acquisition; Methodology; Software; Visualization; Writing – original draft; Writing – review & editing.

## Code and Data Availability Statement

The data, materials, and codes are available on the Open Science Framework website (https://osf.io/y7edf/).

## Supplementary Material



## TECHNICAL TERMS

Semantic cognition: The neural and cognitive mechanisms supporting the acquisition, organization, and flexible retrieval of conceptual knowledge.
Automatic retrieval: Rapid, stimulus-driven access to dominant memory associates with minimal executive control.
Controlled retrieval: Goal-directed memory search requiring executive control processes to inhibit irrelevant information and access weak or nondominant conceptual representations.
Transcranial direct current stimulation (tDCS): A noninvasive method that modulates neural excitability, neuroplasticity, and connectivity using weak electrical currents applied to the scalp.
Associative–Dissociative Task (ADT): A paradigm contrasting automatic free-associative retrieval with controlled retrieval of unrelated concepts.
Associative intrusions: Task-irrelevant associations that arise spontaneously and disrupt coherent cognitive processing and retrieval.
Proactive inhibition: Early suppression of irrelevant memory activations before they enter working memory.
Reactive inhibition: Later suppression of irrelevant memory representations after they have entered working memory.
Reactive inhibition: Later suppression of irrelevant memory representations after they have entered working memory.
