# Supplementary material for: Prefrontal and Cerebellar Contributions to Semantic Memory Retrieval
Source: Neurobiol Lang (Camb). 2026 Mar 26;7:NOL.a.235. doi: 10.1162/NOL.a.235 (PMC13065103; doi:10.1162/NOL.a.235)
Supplement: Supplementary file 1 [file nol-07-235-s001.pdf]

Supplementary Materials

Prefrontal and cerebellar contributions to semantic memory retrieval

Adam Kubinec, Rastislav Rovný, Igor Riečanský, Martin Marko *

* Corresponding authors information: Martin Marko, Institute of Normal and Pathological Physiology, Centre of Experimental Medicine SAS, Sienkiewiczova 1, 813 71 Bratislava, Slovakia (e-mail: martin.marko@savba.sk).

ADT stimuli

Table S1 presents the descriptive statistics for the key psycholinguistic and semantic characteristics of the word stimuli used in the pre-test and post-test blocks of the main experimental task. For detailed information on the procedures used to estimate these characteristics, please refer to the dedicated psychometric validation study [1]. In addition, sensorimotor grounding was estimated using a separate sample of 28 participants (14 males, mean age = 27.46 ± 9.03). The group completed an online sensorimotor rating task, evaluating 178 words based on the relevance of seven sensorimotor features—shape, color, movement, manipulation, sound, smell, and taste—using a 7-point Likert scale (0 = not significant, 6 = highly salient feature). Ratings were averaged for each feature across all words.

Table S1: *Descriptive Statistics for Stimulus Properties in the Pre-test and Post-test Blocks*

Parameter	Pre-test		Post-test		Comparison			
	M	*SD*	*M*	*SD*	*t*	*df*	*p*	d
Word length	5.10	1.11	5.05	1.38	-0.18	74.5	0.858	-0.04
Word frequency	4.82	0.45	4.87	0.37	0.57	75.2	0.573	0.13
Concreteness	4.83	1.51	4.68	1.65	-0.38	67.4	0.705	-0.09
Valence	4.45	0.82	4.36	1.04	-0.42	73.9	0.675	-0.09
Arousal	3.97	0.57	3.91	0.87	-0.43	77.4	0.669	-0.10
Associative typicality	-0.16	1.12	-0.15	1.07	0.06	77.8	0.950	0.01
Sensorimotor grounding	1.82	0.90	1.8	0.85	-0.10	77.8	0.921	-0.02

Note. Welch's t-test was used to compare the stimulus parameters in pre-test and post-test. N = 40 words per block. Word length refers to the number of letters, and frequency to logarithm of word frequency in the national corpus database. Concreteness, valence, and arousal were estimated based on rating scales. Associative typicality is a z-score reflecting whether the word elicits a few dominant responses (high values) or a broader set of more weakly associated response candidates (low values).

Semantic relatedness

Semantic relatedness was evaluated by two independent raters using a six-point ordinal scale (0–5) shown in Table S2. After the experiment, each response was rated by two independent raters on a Likert scale from 'Completely Unrelated' = 0 to 'Prepotent' = 5 (see Table S2 for complete instructions). The final relatedness score was calculated as the average of the two ratings. Raters also screened all responses for errors, which were defined as: (1) violations of the retrieval condition (i.e., FA responses with a relatedness score ≤ 2.5 or DA responses with a score > 2.5), (2) responses that were not common nouns, or (3) unintelligible entries.

Table S2: *Semantic relatedness rating scale*

Relatedness	Rating	Description
Completely unrelated	0	It is very hard to find a meaningful link between the words, even if one tries and considers unlikely themes and/or scenarios. This is considered an excellent dissociation.
Unrelated	1	The stimulus and response are generally unrelated. This is considered a good/default dissociation.
Remote	2	There is only very weak/remote and/or indirect link between the words. This is still considered a sufficient dissociation.
Weakly related	3	Weak or less common association. This is considered a sufficient association.
Related	4	The response is a common association with a clear link to the stimulus. This is considered a good/default association.
Prepotent	5	A strong and dominant association most people deliver. This is considered a prepotent association.

The assessment of rule switching

Rule switching was not experimentally manipulated but examined post hoc from participants' response sequences. Trials were coded as "no switch" when the retrieval condition (e.g., FA) was preceded by the same condition, and as "switch" when it followed the other condition (i.e., DA). On average, participants switched retrieval rules on 52% (SD = 5.5%) of trials in both the baseline block and the tDCS-test block (SDs comparable across blocks). An ANOVA confirmed no significant differences in the proportion of switches between blocks, $F(1,146) = 0.005$, $p = .943$, across stimulation groups, $F(2,146) = 0.936$, $p = .395$, nor in their interaction, $F(2,146) = 0.957$, $p = .386$. The *Switch* factor (yes vs. no) was added in the exploratory analysis (see Supplementary Table S10).

Model specification

The generalized linear mixed-effects models (GLMMs) for validation analyses were specified as follows:

- Validation model 1: RT ~ Retrieval + (1 + Retrieval | ID) + (1 | Stimulus)
- Validation model 2: RT ~ Typicality + (1 + Typicality | ID) + (1 | Stimulus)
- Validation model 3: RT ~ Intrusion + (1 + Intrusion | ID) + (1 | Stimulus)

The GLMMs, used to test the hypotheses, were specified as follows:

- Full model: RT ~ Block * tDCS * Retrieval + (1 + Block + Retrieval | ID) + (1 | Stimulus)
- Typicality model: RT ~ Block * tDCS * Typicality + (1 + Block + Typicality | ID) + (1 | Stimulus)
- Intrusion model: Intrusion ~ Block * tDCS + (1 + Block | ID) + (1 | Stimulus)

The Bayesian mediation analysis was specified as follows:

- Mediator model:

```
brm_med <- brm(INT ~ Block * tDCS + (1 + Block | ID) + (1 | Stimulus),
        data = DATA_ADT_DA, family = bernoulli(),
        iter = 4000, warmup = 1000, chains = 4, cores = 4, seed = 4321,
        control = list(adapt_delta = 0.95))
```

- Outcome model:

```
brm_out <- brm(wRT ~ Block * tDCS + INT + (1 + Block | ID) + (1 | Stimulus),
        data = DATA_ADT_DA, family = Gamma("log"),
        iter = 4000, warmup = 1000, chains = 4, cores = 4, seed = 4321,
        control = list(adapt_delta = 0.95))
```

The exploratory GLMMs were specified as follows:

- Selection model: RT ~ Block * tDCS * Retrieval * Typicality + (1 + Block + Retrieval + Typicality | ID) + (1 | Stimulus)
- LDT RT model: RT ~ Block * tDCS * Word type + (1 + Block + Word type | ID) + (1 | Stimulus)
- Orthographic: RT ~ Block * tDCS * Retrieval + (1 + Block + Retrieval | ID) + (1 | Stimulus)

GLMM outputs

Table S3: *Full GLMM output*

Effect	χ^2	*df*	*p*	
(Intercept)	7638.81	1	<0.001	***
Block	24.05	1	<0.001	***
tDCS	10.99	2	0.004	**
Retrieval	1364.34	1	<0.001	***
Block:tDCS	8.93	2	0.012	*
Block:Retrieval	47.14	1	<0.001	***
tDCS:Retrieval	2.04	2	0.361	
Block:tDCS:Retrieval	11.45	2	0.003	**

Note. *$p < .05$, **$p < .01$, ***$p < .001$.

Table S4: *Associative typicality GLMM output (FA trials only)*

Effect	χ^2	*df*	*p*	
(Intercept)	4380.02	1	<0.001	***
Block	3.09	1	0.079	.
tDCS	7.98	2	0.018	*
Typicality	14.01	1	<0.001	***
Block:tDCS	10.19	2	0.006	**
Block: Typicality	2.28	1	0.131	
tDCS: Typicality	0.48	2	0.787	
Block:tDCS: Typicality	7.36	2	0.025	*

Note. *$p < .05$, **$p < .01$, ***$p < .001$.

Table S5: *Intrusion GLMM output (DA trials only)*

Effect	χ^2	*df*	*p*	
(Intercept)	64.50	1	<0.001	***
Block	0.36	1	0.549	
tDCS	1.34	2	0.511	
Block:tDCS	11.34	2	0.003	**

Note. *$p < .05$, **$p < .01$, ***$p < .001$.

Mediation outputs

Table S6: *Mediation model*

Parameter	Estimate	Est. Error	95% CI Lower	95% CI Upper	$\hat{R}$	Bulk ESS	Tail ESS
Intercept	1.32	0.26	0.83	1.84	1	1988	4024
tDCS-test (vs. baseline)	-0.17	0.18	-0.52	0.18	1	6505	8236
PFC (vs. sham)	-0.04	0.36	-0.75	0.66	1	1665	2931
Interaction (Block x tDCS)	0.52	0.21	0.11	0.93	1	7147	9163

Table S7: *Outcome model*

Parameter	Estimate	Est. Error	95% CI Lower	95% CI Upper	$\hat{R}$	Bulk ESS	Tail ESS
Intercept	1.4	0.04	1.33	1.48	1	2085	3320
tDCS-test (vs. baseline)	-0.09	0.02	-0.14	-0.05	1	8446	9158
PFC (vs. sham)	-0.08	0.05	-0.18	0.02	1	1695	3049
Interaction (Block x tDCS)	0.04	0.03	-0.02	0.09	1	8533	8507
Intrusion	0.24	0.01	0.22	0.26	1	25043	8543

Table S8: *Mediation effects*

Parameter	Median	95% CI	pd (%)	% in ROPE
Indirect Effect	0.12	[0.03, 0.22]	99.27%	29.73%
Direct Effect	0.04	[–0.02, 0.09]	89.53%	100%
Total Effect	0.16	[0.05, 0.27]	99.64%	12.81%
Proportion Mediated %	0.78	[0.37, 1.23]	99.36%	0%

Outputs for exploratory GLMMs

Table S9: *Selection demands GLMM output*

**Effect**	χ^2	*df*	*p*	
(Intercept)	5446.48	1	<0.001	***
Block	28.53	1	<0.001	***
tDCS	7.37	1	0.007	**
Retrieval	938.55	1	<0.001	***
Typicality	6.29	1	0.012	*
Block:tDCS	5.16	1	0.023	*
Block:Retrieval	18.13	1	0.000	***
tDCS:Retrieval	0.44	1	0.509	
Block:Typicality	0.38	1	0.535	
tDCS:Typicality	0.06	1	0.813	
Retrieval:Typicality	9.70	1	0.002	**
Block:tDCS:Retrieval	5.95	1	0.015	*
Block:tDCS:Typicality	0.33	1	0.564	
Block:Retrieval:Typicality	0.45	1	0.504	
tDCS:Retrieval:Typicality	0.06	1	0.805	
Block:tDCS:Retrieval:Typicality	1.94	1	0.163	

Table S10: *Switching demands GLMM output*

**Effect**	χ^2	*df*	*p*	
(Intercept)	5420.03	1	<0.001	***
Block	28.15	1	<0.001	***
tDCS	7.43	1	0.006	**
Retrieval	944.43	1	<0.001	***
Switch	1.59	1	0.207	
Block:tDCS	5.32	1	0.021	*
Block:Retrieval	18.64	1	<0.001	***
tDCS:Retrieval	0.45	1	0.503	
Block:Switch	0.88	1	0.349	
tDCS:Switch	3.56	1	0.059	.
Retrieval:Switch	6.90	1	0.009	**
Block:tDCS:Retrieval	5.87	1	0.015	*
Block:tDCS:Switch	1.16	1	0.282	
Block:Retrieval:Switch	0.16	1	0.691	
tDCS:Retrieval:Switch	0.23	1	0.630	
Block:tDCS:Retrieval:Switch	0.20	1	0.658	

Table S11: *Lexical-decision RT GLMM output*

**Effect**	χ^2	***df***	***p***	
(Intercept)	12425.50	1	<0.001	***
Block	87.61	1	<0.001	***
tDCS	2.41	2	0.300	
LDT	39.45	1	<0.001	***
Block:tDCS	3.96	2	0.138	
Block:LDT	0.66	1	0.418	
tDCS:LDT	2.98	2	0.225	
Block:tDCS:LDT	2.77	2	0.250	
